# Closed-loop assisted versus manual goal-directed fluid therapy during high-risk abdominal surgery: a case–control study with propensity matching

**DOI:** 10.1186/s13054-015-0827-7

**Published:** 2015-03-19

**Authors:** Joseph Rinehart, Marc Lilot, Christine Lee, Alexandre Joosten, Trish Huynh, Cecilia Canales, David Imagawa, Aram Demirjian, Maxime Cannesson

**Affiliations:** Department of Anesthesiology & Perioperative Care, UC Irvine Medical Center, 101 The City Drive South, Orange, CA 92868 USA; Department of Anesthesiology and Intensive Care, Louis Pradel Hospital, Hospices Civils de Lyon, 60 avenue du Doyen Lepine, 69300 Bron, France; Department of Surgery, UC Irvine Medical Center, 101 The City Drive South, Orange, CA 92868 USA

## Abstract

**Introduction:**

Goal-directed fluid therapy strategies have been shown to benefit moderate- to high-risk surgery patients. Despite this, these strategies are often not implemented. The aim of this study was to assess a closed-loop fluid administration system in a surgical cohort and compare the results with those for matched patients who received manual management. Our hypothesis was that the patients receiving closed-loop assistance would spend more time in a preload-independent state, defined as percentage of case time with stroke volume variation less than or equal to 12%.

**Methods:**

Patients eligible for the study were all those over 18 years of age scheduled for hepatobiliary, pancreatic or splenic surgery and expected to receive intravascular arterial blood pressure monitoring as part of their anesthetic care. The closed-loop resuscitation target was selected by the primary anesthesia team, and the system was responsible for implementation of goal-directed fluid therapy during surgery. Following completion of enrollment, each study patient was matched to a non–closed-loop assisted case performed during the same time period using a propensity match to reduce bias.

**Results:**

A total of 40 patients were enrolled, 5 were ultimately excluded and 25 matched pairs were selected from among the remaining 35 patients within the predefined caliper distance. There was no significant difference in fluid administration between groups. The closed-loop group spent a significantly higher portion of case time in a preload-independent state (95 ± 6% of case time versus 87 ± 14%, *P* =0.008). There was no difference in case mean or final stroke volume index (45 ± 10 versus 43 ± 9 and 45 ± 11 versus 42 ± 11, respectively) or mean arterial pressure (79 ± 8 versus 83 ± 9). Case end heart rate was significantly lower in the closed-loop assisted group (77 ± 10 versus 88 ± 13, *P* =0.003).

**Conclusion:**

In this case–control study with propensity matching, clinician use of closed-loop assistance resulted in a greater portion of case time spent in a preload-independent state throughout surgery compared with manual delivery of goal-directed fluid therapy.

**Trial registration:**

ClinicalTrials.gov Identifier: NCT02020863. Registered 19 December 2013

**Electronic supplementary material:**

The online version of this article (doi:10.1186/s13054-015-0827-7) contains supplementary material, which is available to authorized users.

## Introduction

Goal-directed fluid therapy (GDFT) strategies based on cardiac output (CO) optimization have been shown to benefit moderate- to high-risk surgery patients [1-5] and have recently been recommended by professional societies in the United Kingdom [6], France [7] and Europe [8]. However, despite the growing evidence, these strategies are often not implemented in current practice [9]. One of the reasons for this lack of implementation is that GDFT strategies, like any other complex clinical protocol, require significant provider attention and vigilance for consistent implementation, and it is well known that, even under study conditions, protocol compliance rates are often not greater than 50% [10-12].

Computer systems excel at repetitive, “attention-based” tasks and do not suffer from vigilance decrement [13]. For this reason, closed-loop (automated) systems frequently exhibit higher accuracy than clinicians in maintaining a target set point [14], and they have the added benefit of reducing overall provider workload instead of adding to it [15]. On the other hand, *choosing* target set points, or even deciding whether to apply a given clinical protocol to a given patient, is still a judgment-based decision that computer systems are not well suited for. These decisions remain the domain of physicians and other clinical care providers [16]. Ideally, computer-based implementation systems applied under the direction of clinicians could achieve clinical aims by assisting clinicians with the “busy work” of protocol implementation while leaving direction and guidance in the hands of the care providers.

Over the past 4 years, we have developed a closed-loop fluid delivery system designed to assist anesthesia providers with GDFT strategies. The system has been tested extensively in simulation [17,18], engineering studies [19], animal studies [20] and a pilot clinical trial [21]. The aim of the present study was to assess the closed-loop system in a moderate- to high-risk surgical cohort and compare that cohort to matched patients who received manual GDFT. Based on our background research, our hypothesis was that the patients receiving closed-loop assistance would spend more time in a preload-independent state, defined as percentage of case time with stroke volume variation (SVV) less than or equal to 12% [21].

## Materials and methods

### Enrollment

This study was approved by the University of California Irvine (UCI) Institutional Review Board (IRB) (HS 2011-8554) and registered with ClinicalTrials.gov (protocol ID NCT02020863). The study was conducted at UCI Medical Center in Orange, CA, USA, from September 2013 through February 2014. Patients eligible for the study were all those over 18 years of age scheduled for hepatobiliary, pancreatic or splenic surgery and expected to receive intravascular arterial blood pressure monitoring as part of their anesthetic care. Exclusion criteria were under 18 years of age, pregnant, body mass index >35 kg/m^2^, presence of moderate to severe valvular disease, cardiac arrhythmias, left ventricular ejection fraction <40% and right ventricular failure. Additionally, any patients who were found to have metastatic disease during a laparoscopic examination and who had their primary procedures aborted as a result were excluded. Patients were recruited on days when a member of the study group was available to consent and the research staff was available to set up the closed-loop system. In general, this was about 30% of the time during the study period.

As a stipulation of the IRB approval, none of the investigators were permitted to be part of the primary anesthesia team for study cases. All patients included in the study were given details of the project verbally and in writing by a member of the study group, and then they provided written informed consent if willing to participate. The results of this study are reported according to the Strengthening the Reporting of Observational Studies in Epidemiology guidelines [22].

### Study protocol

All of the subjects were patients of one of two surgeons who use similar preoperative preparation protocols (clear fluids only the day before surgery). Bowel preps were not performed in any of the subjects, and all patients followed a strict nothing *per os* after midnight policy for the day of surgery.

Anesthesia was induced with fentanyl (2 μg/kg) and propofol (2 to 3 mg/kg). Following induction, rocuronium was administered to facilitate intubation. Anesthesia was maintained with sevoflurane and fentanyl boluses at the discretion of the anesthesia team. All patients were mechanically ventilated using a volume control mode with tidal volume at 8 ml/kg of ideal body weight and respiratory rate adjusted to achieve an end-tidal CO_2_ between 32 and 36 cmH_2_O. Any necessary adjustments to anesthetic delivery were made at the discretion of the primary anesthesia providers.

All subjects had invasive arterial pressure monitoring via the radial artery planned as part of their anesthetic care. Standard practice in our institution for moderate- to high-risk hepatobiliary surgery patients is to use flow-based monitoring that includes a dynamic predictor of fluid responsiveness such as SVV to provide GDFT. For these cases (both study and control group), all patients were monitored with one of three dedicated EV-1000 monitors and a FloTrac sensor (Edwards Lifesciences, Irvine, CA, USA).

Finally, all patients except those undergoing liver resection had a thoracic epidural placed by the pain service in the preoperative holding area prior to coming to the operating room. The epidural was not activated (save for a 3-ml test dose of 2% lidocaine with 1:200,000 epinephrine) until the surgical case was closing and emergence of the patient was beginning (and data collection completed).

### Closed-loop setup

The closed-loop software (Sironis, Newport Beach, CA, USA) was run on a Shuttle X50 Touchscreen PC (Shuttle Computer Group, City of Industry, CA, USA) running Windows 7 (Microsoft, Redmond, WA, USA). The system was connected via a USB to serial adapter to the serial output port of the EV-1000 for real-time capture of data.

A Q Core Sapphire Multi-Therapy Infusion Pump (Q Core Medical, Netanya, Israel) was used by the closed loop to deliver fluid. The Sapphire pump is a single-channel volumetric pump capable of flow rates from 0.1 to 999 ml per hour. The pump was controlled by the closed-loop system using software provided by Q Core via serial connection (Commands Server R.00).

Following placement of the arterial line (and central line if applicable), but before surgical start time, the closed-loop system target was selected by the primary anesthesia team (scaled stroke volume (SV) increase of 7.5% to 22.5% over 500 ml, standard setting 15%) and started. The primary team was responsible for selection of therapy targets throughout the case (see below in Closed-Loop System Description).

### Closed-loop system description

The closed-loop system has been described extensively in previous publications [17-21]. Briefly, the system monitors SV, heart rate (HR), mean arterial pressure (MAP) and a dynamic predictor of fluid responsiveness such as pulse pressure variation or SVV and uses this information to optimize SV in line with a GDFT protocol. The controller uses both a model layer to formulate a predicted response to a fluid bolus and an adaptive layer for bolus-based error correction during direct fluid management to correct for changes induced by surgical and anesthetic conditions. The final action to be taken by the controller is then determined by a rule-based layer based on data provided by the previous layers [19]. The system is ultimately a slope-seeking controller whose aim is to optimize patients’ fluid status and SV to near the plateau of the Frank–Starling curve. The system delivers 100-ml fluid boluses over the course of 6 minutes when indicated (maximum rate =1,000 ml/hour) and is therefore designed not for high-volume resuscitation, but rather for hemodynamic optimization in line with GDFT protocols.

The exact point on the Frank–Starling curve to be targeted by the controller can be adjusted by the primary anesthesiologist by adjusting the target SV increase, allowing providers to make fluid therapy more liberal or more conservative as appropriate while still following a GDFT protocol. Additionally, the primary anesthesia team can put the system on standby (monitor-only mode), deliver a fluid challenge manually or deliver continuous fluid in the case of an emergency.

The primary anesthesiologist was aware of each bolus of fluid administered by the closed-loop system throughout the surgical procedure. This was done through visual and audio signals alerting the clinician that a fluid bolus was initiated. Additionally, the closed-loop system requires the primary care provider to input the amount of fluid available for delivery, and this volume is limited to 500 ml at a time. This feature was specifically designed as another safety check, as it requires the supervising anesthesiologist to “reauthorize” the system to deliver fluid in 500-ml increments, ensuring the system cannot deliver more than this volume at any time without the supervisor being aware of it. Lower volumes could be chosen at the supervisor’s discretion (200 ml or even 100 ml).

### Fluid administration protocol

**Figure 1:**
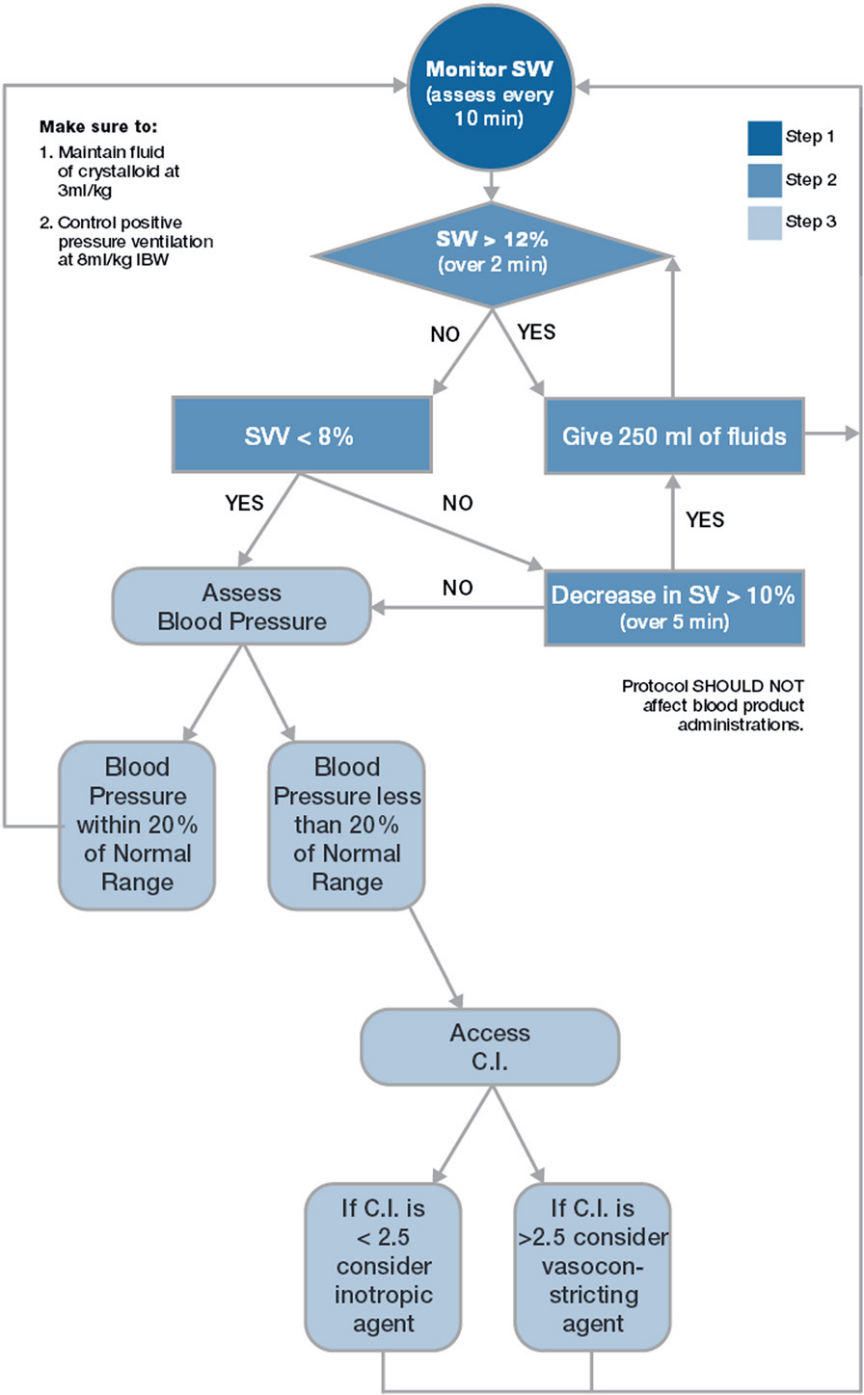
**Manual goal-directed fluid therapy protocol.** The protocol that anesthesia providers utilize in moderate- and high-risk surgery cases at University of California Irvine Medical Center and that was applied to the patients in the manual group. C.I., Cardiac index; IBW, Ideal body weight; SV, Stroke volume; SVV, Stroke volume variation.

In the standard GDFT patients treated at UCI Medical Center, a 3 ml/kg/hr baseline crystalloid infusion is run, and providers follow the protocol shown in Figure 1 by hand [23]. For the closed-loop cases, patients received a restrictive baseline isotonic crystalloid infusion of 3 ml/kg/hr on a pump throughout the duration of the case as maintenance fluid. In addition to the baseline crystalloid, the closed-loop system administered colloids chosen by the primary anesthesia team. Available fluids were 6% hydroxyethyl starch 130/0.4 (Voluven; Fresenius Kabi, Frankfurt, Germany) or 5% serum albumin (Grifols, Sant Cugat del Vallès, Spain). The anesthesia provider had the option of stopping a bolus (or initiating one) if he or she disagreed with the closed-loop fluid management system, and these events were recorded by the system. If the primary anesthesia team felt the patient was fluid-optimized but the patient’s systolic blood pressure was still 20% or more below baseline value (despite appropriate anesthetic depth), they were to consider an inotrope or vasopressor per the lower portion of the manual protocol (Figure 1).

### Case matching

**Figure 2:**
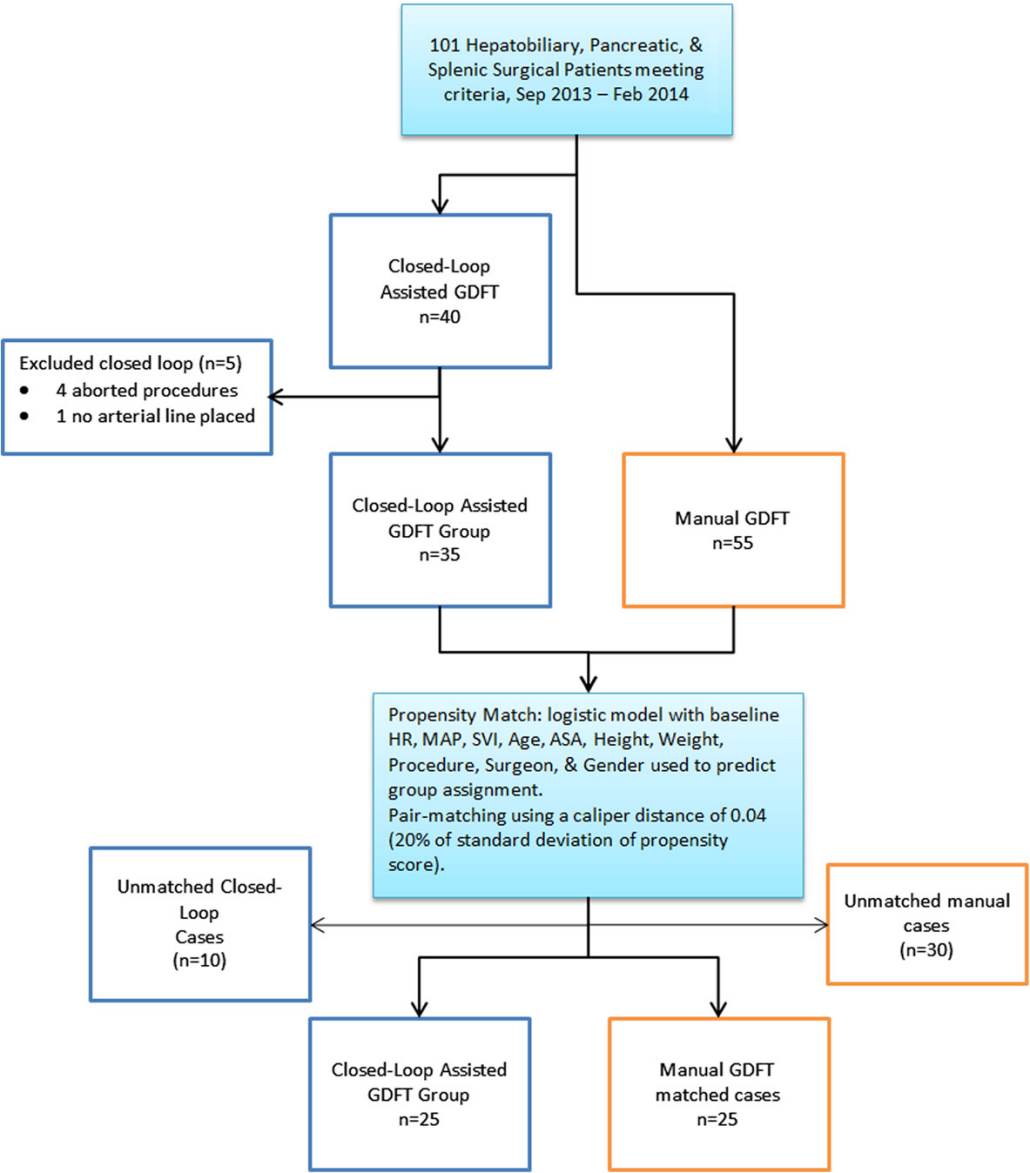
**Recruitment and case-matching process.** Potential subjects were recruited throughout the study period. At the end of recruitment, and after excluding five cases due to aborted procedures or decision to proceed without an arterial line, there were thirty-five study cases in the closed-loop assisted group. During the same time period, 60 other patients who met the inclusion criteria received manual goal-directed fluid therapy (GDFT). All 35 study patients and 60 manual group patients were run in a propensity match process, after which each closed-loop assisted patient was matched to the closest possible manual patient, leaving 35 patients in each group. ASA, American Society of Anesthesiologists Physical Status patient classification; HR, Heart rate; MAP, Mean arterial pressure; SVI, Stroke Volume Index.

Following completion of enrollment, each study patient was matched to a non–closed-loop assisted patient treated during the same time period using a propensity score match to reduce bias [24,25] (Figure 2). The propensity score was estimated using logistic regression to regress group assignment on the predictors in Table 1 (age, height, weight, sex, HR, Stroke Volume Index (SVI), MAP, SVV, specific scheduled procedure and surgeon). Each study patient was then matched 1:1 to a control case using calipers of 0.04% width (20% of the standard deviation (SD) of the propensity score), creating a control manual GDFT (M) group to compare with the closed-loop assisted (CL) group. The complete propensity match process, data table and matched pairs are detailed in Additional file 1.

**Table 1:** **Demographics and baseline hemodynamic data after propensity match** ^**a**^

**Parameter**	**Manual**	**Closed-loop assisted**	Absolute standardized difference (%)*
Age (yr)	61.5 ± 11.4	61.6 ± 12.8	1.3
Height (cm)	166 ± 10	166 ± 12	3.5
Weight (kg)	73.9 ± 17.5	73.9 ± 23.7	0.2
Sex			
Male	11	11	0.0
Female	14	14	
Specific surgical procedure			
Whipple	6	6	0.0
Whipple + additional	6	6	
Distal pancreatectomy	3	3	
Liver resection	8	8	
Complex cholecystectomy	2	2	
ASA Physical Status patient classification			
2	1	1	0.0
3	21	21	
4	3	3	
Baseline hemodynamic data			
Heart rate (bpm)	72 ± 12	73 ± 10	7.0
Mean arterial pressure (mmHg)	88 ± 11	88 ± 10	5.5
Stroke Volume Index	44 ± 12	44 ± 10	1.2
Cardiac Index	3.1 ± 0.9	3.1 ± 0.8	4.8

^a^bpm, Beats per minute. Data are expressed as mean ± standard deviation for scalar data or as counts for categorical data. * Absolute standardized difference >10% is considered a significant imbalance in matching.

### Outcomes, data collection and analysis

The following data were collected continuously at 60-second intervals for each patient by the anesthesia information management system (AIMS) (Surgical Information Systems, Alpharetta, GA, USA): HR, MAP, SV, CO and SVV. Fluid volume, urine output, blood loss, and vasopressors were also recorded in the AIMS by the primary anesthesia team. In study cases, the closed-loop controller also recorded continuous hemodynamic data (HR, MAP, SV and SVV) provided by the EV-1000 at 2-second intervals, as well as fluid administration and provider interactions (set point changes, bolus stops and test boluses administered).

Baseline values for HR and MAP were taken from the preoperative anesthesia assessment. Baseline values for SVI and Cardiac Index (CI) were taken as the mean of the first minute’s recording 5 minutes after placement of the arterial line and calibration of the hemodynamic monitoring system. Mean case value for each hemodynamic variable was calculated as the average value of all recordings between baseline and the beginning of surgical closure. Final case value for each hemodynamic variable was defined as the average value of each parameter over the last minute before the beginning of emergence (lowering of anesthetic agent in preparation for extubation). Finally, a Postoperative Morbidity Survey (POMS) score [26] was calculated for each patient, and length of Surgical Intensive Care Unit (SICU) and hospital stay were recorded.

The primary outcome between groups was GDFT compliance, defined as percentage of case time where SVV was ≤12%. Upon study initiation in September 2012, the original endpoint was cardiac SV. When our case series in Europe was finished, informal comparison with other standard case data showed strong effects on SVV for the CL group, but weaker effects on SV itself [21]. Moreover, we observed that SVI as recorded by the minimally invasive CO monitoring systems has a very wide distribution between and within patients [27,28], meaning a large sample size would be needed to detect true effects on this variable. Second, Pearse and colleagues reported the full “Optimisation of peri-operative cardiovascular management to improve surgical outcome” (OPTIMISE; ISRCTN04386758) study in 2014 [5]. One of the findings of that study was the importance of protocol compliance to the beneficial effects of the GDT:

In the prespecified adherence-adjusted analysis conducted using established methods [*citation omitted*], the observed treatment effect was strengthened when the 65 patients whose care was nonadherent [*internal cross-reference omitted*] were assumed to experience the same outcome as if they had been allocated to the alternative group (RR, 0.80; 95% CI, 0.61-0.99; *P* = .04). (p 2186 [5])

On the basis of the pilot study data, the findings derived from the OPTIMISE trial and the fact that it would be the same endpoint as the pilot study (GDFT protocol compliance), we decided to treat protocol compliance measured as SVV as the primary outcome measure in the present study.

Secondary endpoints were case mean hemodynamic values, final case hemodynamic values, fluid volumes, urine output, postoperative complications, SICU length of stay and hospital length of stay. Additional measures of controller performance recorded during the study were distribution of fluid boluses given across cases (minimum, maximum, mean and SD), percentage of the boluses that resulted in an SV increase and predictive specificity as number of fluid boluses given to a patient increased. Provider interactions with the system were also recorded (start and stop of boluses and changes in controller set point).

Based on local data, case time with SVV ≤12 in the case types to be studied was approximately 80 ± 10% over the previous 1-year period, or about 20 ± 10% non-compliance time. Cutting non-compliance time in half (or more) was considered a clinically relevant endpoint that, assuming a power of 0.8 and a significance level of 0.05, would require 21 patients in each group to be adequately powered.

Continuous scalar data were tested for normality using a Kolmogorov–Smirnov test. If normal, the data are reported as mean ± SD and comparisons were made using Student’s *t*-test. Non-normally distributed scalar data and ordinal data (namely, POMS scores) are reported as median (25th, 75th percentile), and comparisons were made with the Mann–Whitney *U* test. The significance level for the primary outcome was set at 0.05. All other comparisons were made at the 0.025 level to compensate for the multiple secondary endpoints.

## Results

### Enrollment and case matching

Forty patients were enrolled in the study over the study period, out of a total of one hundred one patients who met the eligibility criteria. Four were excluded when surgery was aborted because of discovery of metastatic disease on initial laparoscopic examination, and one patient was excluded because the primary team chose not to place an arterial line after enrollment.

The remaining 35 patients were matched 1:1 against the control patients using the propensity score. Of the 61 control patients, 6 had metastatic disease or did not have CO monitoring during their procedures, leaving 55 completed controls that were performed during the same time period. A total of 25 matched pairs were found within the predefined caliper distance. Demographic data for the two groups, as well as baseline hemodynamic values (HR, SVI and MAP) and specific procedure distribution, are shown in Table 1. Absolute standardized distance for each match parameter was <10%, suggesting the propensity score matching process was effective in selecting a control group similar to the study group at baseline [29].

### Case variables

There was no significant difference in case time, estimated blood loss (EBL), urine output (UO), total crystalloid administration, total colloid administration, crystalloid measured as milligrams per kilogram per hour, colloid as milligrams per kilogram per hour, transfusion rates or administration of cardiovascular support agents (Table 2) between groups.

**Table 2:** **Hemodynamic and case data** ^**a**^

	**Manual**	**Closed-loop**	***P*** **-value**
Case time (min)	472 (346, 571)	433 (324, 583)	0.467
Estimated blood loss (ml)	200 (50, 400)	100 (25, 250)	0.182
Urine output (ml)	525 (345, 705)	625 (300, 900)	0.491
Total crystalloid (ml)	2500 (1,567, 3,817)	2100 (1,554, 3,653)	0.628
Crystalloid (ml/kg/hr)	4.9 (3.6, 6.4)	4.6 (3.5, 6.8)	0.977
Total colloid (ml)	750 (375, 1,050)	576 (400, 1,199)	0.938
Colloid (ml/kg/hr)	1.4 (0.7, 1.7)	1.4 (0.8, 1.6)	0.634
Blood (U/case)	0 (0, 0)	0 (0, 0)	0.760
Phenylephrine (μg)	300 (50, 700)	200 (0, 500)	0.480
Ephedrine (mg)	10 (5, 25)	10 (0, 20)	0.510
Length of SICU stay (d)	1 (0, 1)	0 (0, 2)	0.648
Length of hospital stay (d)	6 (4, 7)	6 (3, 9)	0.652
Total length of stay (d)	7 (5, 8)	6 (4, 11)	0.815
Mean HR (bpm)	78 ± 10	72 ± 10	0.035
Mean SVI (ml/m^2^)	43 ± 9	45 ± 10	0.615
Mean CI (L/m^2^/min)	3.5 ± 0.8	3.3 ± 0.8	0.373
Mean MAP (mmHg)	83 ± 9	79 ± 8	0.077
Mean SVR (dyn · s/cm^5^)	1070 ± 302	1121 ± 353	0.580
Mean SVV (%)	8.8 ± 2.5	7.0 ± 1.2	**0.003** ^†^
End HR (bpm)	88 ± 13	77 ± 10	**0.003** ^†^
End SVI (ml/m^2^)	42 ± 11	45 ± 11	0.308
End CI (L/m^2^/min)	3.4 ± 1.1	3.4 ± 0.9	0.811
End MAP (mmHg)	82 ± 13	78 ± 19	0.411
End SVR (dyn · s/cm^5^)	1042 ± 336	1033 ± 315	0.926
End SVV (%)	9.2 ± 3.1	6.9 ± 3.4	0.015*
POMS score			
Postoperative day 3	3 (2,4)	3 (2,4)	0.683
Postoperative day 5	1 (1,3)	2 (1,3)	0.408

^a^bpm, Beats per minute; CI, Cardiac Index; HR, Heart rate; MAP, Mean arterial pressure; POMS, Postoperative Morbidity Survey; SICU, Surgical Intensive Care Unit; SVR, Systemic vascular resistance; SVI, Stroke Volume Index; SVV, Stroke volume variation. Data are presented as mean ± standard deviation for normally distributed scalar data, or as median (25th, 75th percentiles) for non-normally distributed scalar or ordinal data. *Primary outcome tested at *P* <0.05. ^†^Secondary outcome tested at *P* <0.025.

### Primary outcome

**Figure 3:**
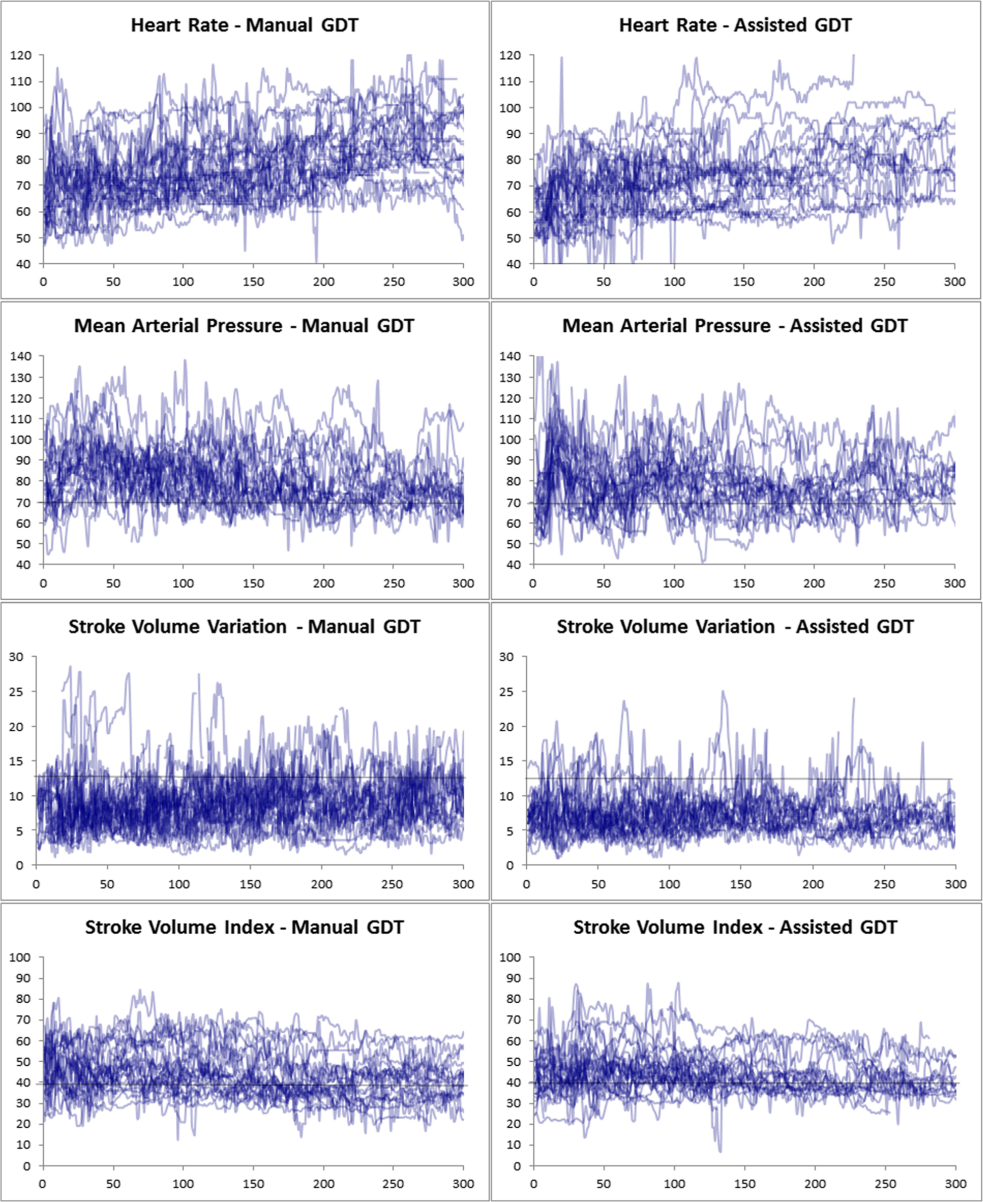
**Hemodynamics for the first 5 hours of all cases.** Each graph is an overlay of all 25 patients in the group. Only the first 300 minutes of cases (if longer than 5 hours) are shown.

The CL group spent a significantly higher portion of case time in a preload-independent state. The average CL patient spent 95 ± 6% of the case time with an SVV ≤12%, whereas the average M group patient spent 87 ± 14% of the case time in this range (*P* =0.008). Both of these rates were significantly higher than the general rate recorded in the previous year (80%). Box plots for compliance in both groups are shown in Figure 3.

### Secondary outcomes

The CL group had a significantly lower case mean SVV compared with the M group (7.7 ± 12 versus 8.8 ± 2.5, *P* =0.003). There was no significant difference in case mean HR, SVI, CI, MAP or SVI, though there was a trend toward lower HR in the CL group (non-significant at *P* =0.035 because of the significance level adjustment for multiple outcomes). The CL group also had a lower final SVV (6.9 ± 3.4 versus 9.2 ± 3.1, *P* =0.015) and a lower final HR compared with the M group (77 ± 12 versus 88 ± 13, *P* =0.003). There was no significant difference in final CI, MAP or systemic vascular resistance between groups.

**Figure 4:**
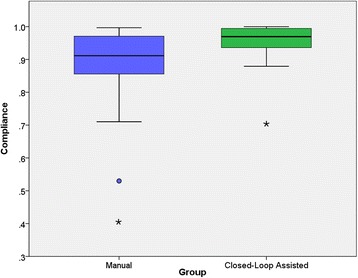
**Goal-directed fluid therapy compliance by group.** Box plots for the manual and closed-loop assisted groups, including outliers, are shown. The closed-loop assistance not only improved mean compliance to goal-directed fluid therapy (GDT) principles but also substantially reduced the variability in compliance within the group (manual group compliance standard deviation (SD) =14%, closed-loop group compliance SD =6%). Asterisks represent outliers, dot represent extreme outliers.

There were no significant differences in length of SICU stay, total hospital stay, postoperative day 3 POMS score or postoperative day 5 POMS score. Case variables, hemodynamic data, length of stay data and POMS scores are all shown in Table 2. Principal hemodynamic variables for all cases are graphed in Figure 4.

### Controller actions

The closed-loop system gave a total of 214 discrete 100-ml fluid boluses across the 25 study cases, with an average of 8.5 ± 6.5 boluses per case. The minimum given in a case was one bolus, and the maximum was twenty-six. Of the 214 fluid boluses given by the controller, 137 resulted in a scaled cardiac SV increase of at least 15% over 500 ml, and 124 resulted in a scaled SV increase of at least 10% over 250 ml. No comparison data are available for the control group, as individual fluid boluses were not recorded by the providers in the AIMS.

The controller’s predictive specificity improved with increased experience with the patients. The first to third fluid boluses given to patients had a mean scaled SVI increase of 10.2 ± 43%, the fourth to seventh 12 ± 36% and the eight and later 18 ± 35%.

In terms of interactions with the closed-loop system, providers delivered 18 fluid boluses through the closed-loop system by hand. The mean scaled SVI increase following provider boluses was 6.8 ± 28.4%, and the mean scaled increase following system boluses was 14 ± 29%, though this difference was non-significant due to the wide ranges.

Thirty-four fluid boluses that the system initiated were halted by providers before or during delivery. None of the providers put the system into a continuous infusion mode during use. In terms of the system targets, across all cases, the system was set to use the default 15% SV increase target for 92% of case time. It was set to 11% (more liberal) 4.5% of the time, and 3.5% of the time it was set to 19% (more conservative). The system was never set to the 7.5% or 22.5% targets at the edges of the available range.

## Discussion

In this article, we report the first comparison of a closed-loop fluid administration system to assist clinicians in providing GDFT versus manual GDFT application in surgical patients. The 25-patient cohort was compared with a propensity score-matched control group and was found to have spent a significantly greater portion of the case time in a preload-independent state (case time with SVV ≤12%) and to have a lower mean HR throughout surgery. There were no significant differences in fluid administration volume, EBL or other case variables between groups.

This trial is the first in which the quality of GDFT delivery by the closed-loop system has been tested in a true clinical environment. Moreover, the system was compared with a provider cohort well-versed in GDFT principles, having had a clinical protocol in place since 2011 mandating use of advanced hemodynamic monitoring and SV optimization in hepatobiliary surgery and hosting an annual conference focusing on GDFT principles.

The finding that the CL group spent more of the case time with cardiac SV in a preload-independent state (and with a lower average HR) suggests that this group was well-resuscitated in terms of intravascular volume. One would expect this to be readily accomplished simply by heavily volume-loading patients. In this study, however, and in contrast to the differences in SVV and HR, fluid administration volumes were virtually identical between groups, as was EBL. Given the absence of any other obvious baseline or case difference to explain the SVV difference between the groups (Tables 1 and 2), this difference seems most likely to be a result of the timing of fluid administration. This finding may have been predicted by our previous simulation and animal studies [17,20] in which similar results were found to arise from differences in timing of administration between groups.

The CI in both groups increased by about 10% over the duration of the cases, likely as a response to the physiologic stress of surgery. In the CL group, this increase occurred through a combination of HR and SV increases (Tables 1 and 2), whereas in the M group, the CI increase resulted more from the HR increase alone. The mean SVI in the M group was actually lower at the end of the cases than at the beginning, whereas in the CL group, it increased modestly throughout, though these differences were not statistically significant.

On the basis of the results of this initial study, the closed-loop system may benefit surgical care in several ways. First, use of the closed-loop system should provide high-quality implementation of GDFT with minimal additional clinician workload compared with non-GDFT practice. From a quality assurance standpoint, the closed-loop system could also help standardize GDFT delivery across providers and even institutions. Finally, if the system is shown to reduce overall provider “tasks” with regard to fluid therapy (for example, starting a bolus, calculating change in SV or tracking fluid delivered), this would free up time and attention to focus on higher-level care factors.

This study provides the first clinical evidence that this closed-loop fluid administration system can assist clinicians in providing, and even improving, the quality of GDFT. A number of important study questions remain. Validation of these results at other centers and in randomized studies are warranted, as are studies in other types of surgical cases. Comparison of the closed-loop assistance in this study was to standard hand-provided GDFT; comparison to other fluid management strategies, especially in institutions without guidelines, would be valuable. Moreover, studies of provider workload with versus without the closed-loop system could also be informative, as would other studies specifically looking at patient outcomes in the postoperative period. Finally, the potential for applications outside the intraoperative period (for example, in the SICU) could be investigated.

### Limitations

There are several limitations of the present study that warrant discussion. The most obvious limitation of the study is that it was not randomized, and the case matching may have failed to correct an unrecognized bias in one of the groups. The use of the propensity matching method (versus a standard case match based on a small subset of variables) was one attempt to control for this possibility, and, although the baseline group data suggest that the groups were indeed similar on the compared characteristics, the potential for bias cannot be completely eliminated.

With regard to the regression done for the propensity score, it is possible that, although patients with moderate to severe cardiac disease were excluded from the study, baseline differences existed between the study and control groups, and these were not accounted for in the model. Also, the presence of the research team and closed-loop system in the study group may have led to behavioral changes among the primary anesthesia care team that may have affected outcomes.

CO and SV were measured from a peripheral arterial line, a technology with known limitations. Both groups used the same monitoring system, however, and, given the declining use of Swan-Ganz catheters in general practice, the majority of GDFT worldwide is being applied via the same or comparable devices. The highly accessible nature of minimally invasive CO monitoring was a large part of what made intraoperative closed-loop GDFT feasible in the first place [30].

Finally, arterial line dampening is checked for within the closed-loop algorithm, and an alert is issued to the supervisor if dampening is suspected. Arterial line dampening was not independently checked for in the control patients; this was dependent on the managing anesthesiologist. If arterial line dampening occurred and went uncorrected for a significant portion of case time, this may have affected results.

## Conclusions

In this case–control study with propensity matching, clinician use of closed-loop assistance resulted in a greater portion of case time spent in a preload-independent state throughout surgery compared with manual delivery of GDFT, as well as a lower final HR as compared with manual administration. SVI, MAP and cardiac index were not significantly different between groups.

## Key messages

- Closed-loop assistance with intraoperative goal-directed fluid therapy resulted in greater case time with patients in a preload-independent state.
- There were no significant differences in fluid administration volume between groups.
- The closed-loop hemodynamic optimization and fluid management required minimal provider supervision after therapy targets were set.
- Closed-loop hemodynamic optimization and fluid management may enable consistent adherence to perioperative goal-directed therapy protocols.

## Additional file

Additional file 1:
**Detailed description of propensity matching process as well as raw matching table and data.**

## Abbreviations

AIMS: Anesthesia information management system
ASA: American Society of Anesthesiologists
bpm: Beats per minute
CI: Cardiac Index
CL: Closed loop group
CO: Cardiac output
EBL: Estimated blood loss
GDFT: Goal-directed fluid therapy
HR: Heart rate
IRB: Institutional Review Board
M: Manual control group
MAP: Mean arterial pressure
POMS: Postoperative Morbidity Survey
SD: Standard deviation
SICU: Surgical Intensive Care Unit
SV: Stroke volume
SVI: Stroke Volume Index
SVR: Systemic vascular resistance
SVV: Stroke volume variation
UCI: University of California Irvine
UO: Urine output
