# Supplementary material for: Closed-loop assisted versus manual goal-directed fluid therapy during high-risk abdominal surgery: a case–control study with propensity matching
Source: Crit Care. 2015 Mar 19;19(1):94. doi: 10.1186/s13054-015-0827-7 (PMC4372998; doi:10.1186/s13054-015-0827-7)
Supplement: Additional file 1: — Detailed description of propensity matching process as well as raw matching table and data. [file 13054_2015_827_MOESM1_ESM.docx]

**Propensity Matching Details**

Following completion of enrollment each study patient was matched to a non-closed-loop assisted case performed during the same time period using a propensity score match to reduce bias. The figure below depicts the recruitment and case matching process. Details of the propensity match are provided in this electronic supplement.

**
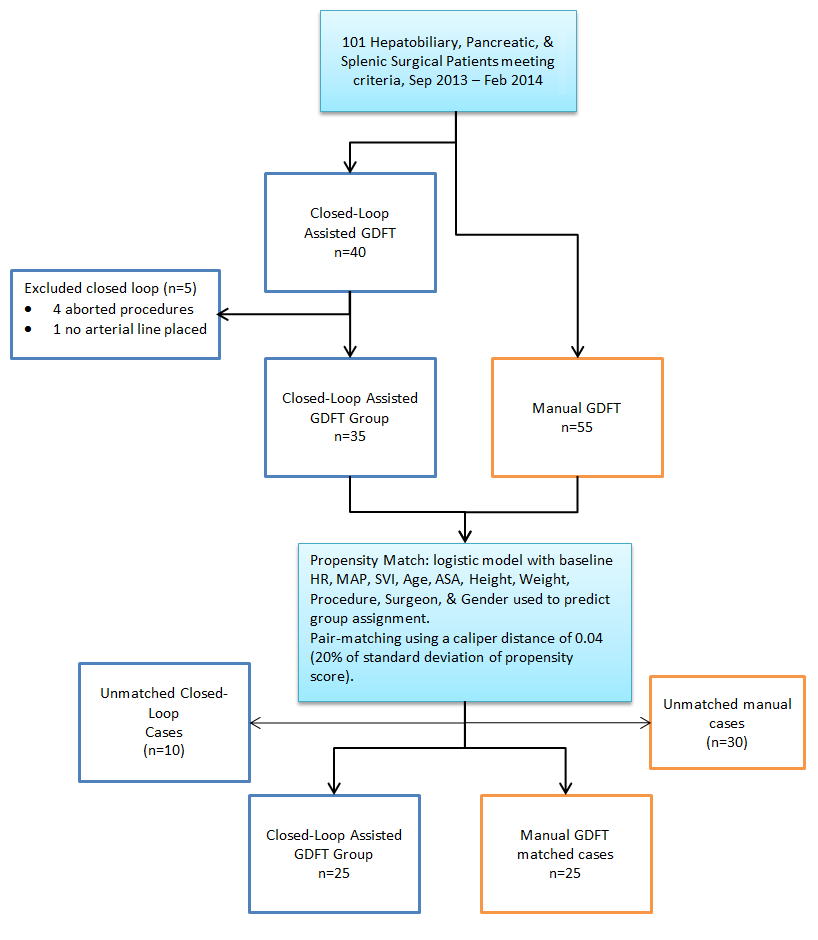
**

Figure 1: Recruitment flowchart

**Parameter Selection**

The parameters chosen for inclusion in the case match process are generally selected based on their high likelihood of having an influence on the study outcome of interest. For this study, the outcomes of interest were hemodynamic and outcome related. As such, the most important pre-surgical parameters to include in the match would be baseline hemodynamic values and the type of surgery the patient was having. Baseline heart rate and mean arterial pressure were taken from the preoperative anesthesia module from the vital signs collected in preop holding before entering the OR. Baseline value for SVI and CI were taken as the mean of the first minute’s recording 5 minutes after placement of the arterial line and initiation of the hemodynamic monitoring system (FloTrac, Edwards Lifesciences, Irvine CA).

In addition to baseline hemodynamics and case type, several other factors were included. Specific surgeon was included as the two surgeons who did the cases in this study had different average case times and surgical techniques, so this may have been an independent predictor. Patient specific factors that may have been independent predictors were also included: height, weight, gender, and age.

Finally, American Society of Anesthesiologists patient score is a recognized independent risk predictor for overall patient health, so this was also included in the match. Patient disease states that would have directly contributed to outcome differences in the primary variables of interest like cardiovascular or pulmonary disease were rare or mild when present because of the exclusion criteria of the study, for this reason adding match criteria for specific organ system diseases was not deemed necessary in addition to the composite ASA score.

**Table 1 – Demographics and Baseline Hemodynamic Data After Propensity Match**

| **Parameter** | | **Manual** | **Closed-Loop Assisted** | **Absolute Standardized Difference (%)*** |
| --- | --- | --- | --- | --- |
| Age (y) | | 61.5 ± 11.4 | 61.6 ± 12.8 | 1.3 |
| Height (cm) | | 166 ± 10 | 166 ± 12 | 3.5 |
| Weight (kg) | | 73.9 ± 17.5 | 73.9 ± 23.7 | 0.2 |
|  |  |  |  |  |
| **Gender** | |  |  |  |
|  | Male | 11 | 11 | 0.0 |
|  | Female | 14 | 14 |  |
| **Specific Procedure** | |  |  |  |
|  | Whipple | 6 | 6 | 0.0 |
|  | Whipple + additional | 6 | 6 |  |
|  | Distal pancreatectomy | 3 | 3 |  |
|  | Liver resection | 8 | 8 |  |
|  | Complex cholecystecomy | 2 | 2 |  |
| **ASA Patient Classification** | |  |  |  |
|  | 2 | 1 | 1 | 0.0 |
|  | 3 | 21 | 21 |  |
|  | 4 | 3 | 3 |  |
|  |  |  |  |  |
| **Baseline Hemodynamic Data** | |  |  |  |
|  | Heart rate (bpm) | 71.8 ± 11.6 | 72.6 ± 10.0 | 7.0 |
|  | Mean arterial pressure (mmHg) | 88.3 ± 10.7 | 87.7 ± 10.2 | 5.5 |
|  | Stroke volume index | 43.7 ± 11.7 | 43.5 ± 10.3 | 1.2 |
|  | Cardiac Index | 3.11 ± 0.85 | 3.12 ± 0.83 | 4.8 |

*Data expressed as mean ± standard deviation for scalar data or expressed as counts for categorical data.*

** Absolute standardized difference > 10% is considered a significant imbalance in matching.*

**Model**

The propensity score was estimated using logistic regression to regress group assignment on the predictors in Table 1 (shown above). A generalized linear model with a binary logistic response predicting group assignment was run using the variables listed in the table as factors and covariates as appropriate. Only main effects of each predictor were used with closed-loop patients assigned the score of 1 and manual patients assigned 0. The mean propensity score for the closed-loop assigned patients was 0.52 ± 0.20 and for the manual group 0.35 ± 0.17.

Each study patient was then matched 1:1 to a control case using calipers of 0.04 width (20% of the standard deviation of the propensity score) creating a control manual GDFT group (M) to compare to the closed-loop assisted group (CL). Matches that minimized residual differences were favored over nearest propensity score matches so long as they were within the caliper distance. The mean match distance for propensity score across all matched patient pairs was 0.015 ± 0.012. The absolute standardized differences[^1^](#_ENREF_1)^,^ [^2^](#_ENREF_2) are shown in Table 1.

The complete dataset used for matching along with the propensity score, predicted group assignment, and matched patient pairs are shown below in Table 2.

More complex regression modeling (using nested or hierarchical structures for the predictor variables) were not used because it was felt they would complicate modeling without substantially reducing the limitations inherent in case matching study.

**Table 2 – Case Matches**

| SVI | HR | MAP | ASA | Kg | PROC | AGE | SURGEON | GROUP | PROPENSITY SCORE | PREDICTED GROUP | CASE MATCH PAIR |
| --- | --- | --- | --- | --- | --- | --- | --- | --- | --- | --- | --- |
| 38 | 116 | 126 | 3 | 97 | 4 | 62 | 1 | M | 0.03 | M |  |
| 35 | 80 | 101 | 3 | 70 | 4 | 74 | 1 | C | 0.10 | M | 1 |
| 31 | 102 | 100 | 3 | 59 | 4 | 63 | 1 | M | 0.10 | M |  |
| 36 | 82 | 105 | 3 | 54 | 1 | 67 | 1 | M | 0.12 | M |  |
| 33 | 70 | 95 | 3 | 57 | 4 | 77 | 2 | M | 0.13 | M | 1 |
| 38 | 74 | 100 | 3 | 44 | 1 | 75 | 2 | M | 0.14 | M |  |
| 38 | 65 | 96 | 3 | 62 | 4 | 73 | 1 | M | 0.16 | M |  |
| 44 | 62 | 98 | 3 | 48 | 4 | 69 | 1 | M | 0.17 | M |  |
| 31 | 117 | 103 | 3 | 57 | 3 | 41 | 1 | M | 0.17 | M |  |
| 31 | 70 | 90 | 3 | 59 | 4 | 75 | 2 | C | 0.17 | M | 2 |
| 40 | 60 | 89 | 3 | 70 | 1 | 71 | 1 | M | 0.18 | M |  |
| 44 | 114 | 89 | 3 | 83 | 5 | 63 | 1 | M | 0.20 | M |  |
| 39 | 77 | 81 | 3 | 48 | 4 | 77 | 2 | M | 0.21 | M | 2 |
| 35 | 52 | 92 | 3 | 76 | 4 | 78 | 1 | M | 0.21 | M | 3 |
| 44 | 80 | 99 | 3 | 73 | 1 | 67 | 2 | M | 0.21 | M |  |
| 32 | 88 | 95 | 3 | 59 | 3 | 64 | 1 | C | 0.22 | M | 3 |
| 31 | 72 | 92 | 2 | 60 | 3 | 65 | 2 | M | 0.23 | M |  |
| 40 | 79 | 90 | 3 | 88 | 4 | 67 | 1 | M | 0.23 | M | 4 |
| 39 | 75 | 97 | 3 | 77 | 4 | 52 | 1 | M | 0.24 | M |  |
| 50 | 92 | 88 | 3 | 62 | 4 | 58 | 1 | M | 0.24 | M |  |
| 33 | 55 | 101 | 3 | 87 | 4 | 56 | 1 | M | 0.24 | M |  |
| 36 | 68 | 95 | 3 | 64 | 1 | 66 | 2 | M | 0.25 | M |  |
| 38 | 64 | 97 | 3 | 78 | 1 | 67 | 1 | M | 0.25 | M |  |
| 43 | 75 | 91 | 3 | 73 | 4 | 60 | 1 | M | 0.25 | M |  |
| 47 | 77 | 97 | 3 | 78 | 1 | 68 | 2 | C | 0.26 | M | 4 |
| 33 | 62 | 100 | 3 | 76 | 5 | 54 | 1 | M | 0.27 | M | 5 |
| 38 | 71 | 90 | 3 | 60 | 3 | 73 | 1 | C | 0.30 | M | 5 |
| 47 | 79 | 84 | 3 | 76 | 4 | 66 | 1 | C | 0.30 | M | 6 |
| 25 | 91 | 88 | 3 | 143 | 4 | 55 | 1 | M | 0.31 | M |  |
| 33 | 88 | 74 | 3 | 61 | 1 | 71 | 2 | M | 0.32 | M |  |
| 42 | 67 | 78 | 2 | 61 | 4 | 56 | 2 | M | 0.33 | M | 6 |
| 46 | 56 | 103 | 3 | 109 | 3 | 77 | 1 | M | 0.33 | M |  |
| 45 | 75 | 107 | 3 | 94 | 3 | 51 | 1 | M | 0.34 | M | 7 |
| 43 | 64 | 92 | 3 | 57 | 3 | 71 | 1 | C | 0.34 | M | 7 |
| 39 | 53 | 94 | 3 | 73 | 4 | 54 | 2 | C | 0.35 | M | 8 |
| 32 | 60 | 88 | 3 | 77 | 4 | 51 | 1 | M | 0.35 | M |  |
| 35 | 71 | 81 | 3 | 62 | 3 | 78 | 1 | M | 0.36 | M | 8 |
| 31 | 55 | 93 | 3 | 75 | 3 | 70 | 1 | M | 0.36 | M | 9 |
| 46 | 89 | 81 | 3 | 73 | 1 | 63 | 1 | C | 0.36 | M | 9 |
| 41 | 85 | 107 | 4 | 62 | 1 | 77 | 2 | C | 0.36 | M | 10 |
| 31 | 91 | 79 | 3 | 82 | 5 | 58 | 2 | M | 0.36 | M | 10 |
| 38 | 77 | 86 | 3 | 75 | 1 | 59 | 2 | M | 0.36 | M | 11 |
| 61 | 58 | 91 | 3 | 110 | 4 | 74 | 1 | C | 0.39 | M | 11 |
| 63 | 73 | 94 | 3 | 19 | 2 | 62 | 1 | M | 0.41 | M | 12 |
| 28 | 82 | 74 | 3 | 97 | 1 | 63 | 1 | C | 0.43 | M | 12 |
| 54 | 73 | 80 | 3 | 66 | 1 | 67 | 1 | M | 0.43 | M | 13 |
| 65 | 85 | 87 | 2 | 43 | 1 | 30 | 1 | C | 0.44 | M | 13 |
| 44 | 78 | 80 | 3 | 69 | 5 | 60 | 2 | C | 0.44 | M | 14 |
| 52 | 68 | 95 | 3 | 47 | 2 | 60 | 1 | C | 0.44 | M | 15 |
| 43 | 65 | 87 | 3 | 61 | 2 | 73 | 1 | M | 0.45 | M | 14 |
| 31 | 92 | 63 | 3 | 118 | 4 | 70 | 1 | M | 0.45 | M | 15 |
| 23 | 62 | 94 | 3 | 93 | 5 | 33 | 1 | C | 0.45 | M | 16 |
| 40 | 68 | 79 | 3 | 60 | 5 | 55 | 1 | M | 0.46 | M | 16 |
| 47 | 78 | 92 | 3 | 67 | 3 | 48 | 1 | C | 0.46 | M | 17 |
| 27 | 78 | 86 | 3 | 143 | 2 | 77 | 1 | M | 0.48 | M | 17 |
| 56 | 72 | 100 | 3 | 55 | 2 | 47 | 1 | M | 0.49 | M | 18 |
| 48 | 77 | 87 | 3 | 98 | 3 | 61 | 1 | C | 0.50 | C | 18 |
| 36 | 68 | 103 | 4 | 86 | 4 | 62 | 1 | C | 0.51 | C | 19 |
| 44 | 82 | 83 | 3 | 54 | 2 | 56 | 1 | M | 0.52 | C |  |
| 55 | 67 | 91 | 3 | 86 | 3 | 60 | 1 | M | 0.53 | C | 19 |
| 40 | 58 | 78 | 3 | 79 | 3 | 74 | 1 | C | 0.53 | C | 20 |
| 55 | 68 | 79 | 3 | 89 | 1 | 65 | 1 | M | 0.54 | C | 20 |
| 54 | 63 | 108 | 4 | 73 | 4 | 62 | 1 | M | 0.54 | C |  |
| 32 | 78 | 90 | 4 | 77 | 4 | 64 | 1 | M | 0.57 | C |  |
| 65 | 64 | 76 | 3 | 97 | 4 | 66 | 1 | C | 0.57 | C | 21 |
| 55 | 82 | 101 | 4 | 83 | 1 | 71 | 1 | M | 0.58 | C | 21 |
| 46 | 72 | 84 | 3 | 83 | 2 | 60 | 1 | C | 0.60 | C | 22 |
| 55 | 57 | 99 | 4 | 62 | 4 | 69 | 1 | M | 0.60 | C | 22 |
| 49 | 62 | 85 | 3 | 88 | 2 | 67 | 1 | C | 0.61 | C | 23 |
| 40 | 68 | 102 | 4 | 77 | 1 | 63 | 2 | M | 0.62 | C | 23 |
| 32 | 69 | 87 | 3 | 73 | 2 | 36 | 2 | C | 0.67 | C |  |
| 43 | 75 | 91 | 4 | 69 | 4 | 57 | 2 | C | 0.67 | C |  |
| 47 | 67 | 74 | 3 | 47 | 1 | 36 | 2 | C | 0.67 | C | 24 |
| 64 | 76 | 96 | 4 | 71 | 1 | 75 | 2 | C | 0.67 | C |  |
| 52 | 64 | 91 | 3 | 82 | 2 | 44 | 1 | C | 0.68 | C |  |
| 50 | 74 | 79 | 3 | 56 | 5 | 19 | 1 | C | 0.70 | C |  |
| 71 | 69 | 83 | 3 | 51 | 3 | 46 | 2 | M | 0.71 | C | 24 |
| 27 | 59 | 95 | 4 | 82 | 2 | 67 | 1 | C | 0.77 | C |  |
| 46 | 57 | 102 | 4 | 129 | 3 | 73 | 1 | C | 0.78 | C |  |
| 36 | 93 | 80 | 4 | 118 | 1 | 58 | 1 | C | 0.80 | C |  |
| 51 | 60 | 82 | 4 | 58 | 4 | 54 | 1 | C | 0.81 | C |  |
| 38 | 80 | 62 | 4 | 78 | 4 | 63 | 1 | C | 0.84 | C | 25 |
| 52 | 67 | 70 | 3 | 67 | 3 | 29 | 2 | M | 0.85 | C | 25 |
| 57 | 64 | 94 | 4 | 70 | 2 | 56 | 1 | C | 0.87 | C |  |

*Procedures: 1-whipple, 2-distal pancreas or spleen, 3-whipple+chole or liver, 4-Liver resection, 5 – gallbladder and lymph nodes, biliary tree, etc. Group: 0-manual, 1-closed-loop.*

**REFERENCES**

1. P. C. Austin, *Goodness-of-fit diagnostics for the propensity score model when estimating treatment effects using covariate adjustment with the propensity score.* Pharmacoepidemiol Drug Saf, 2008. **17**(12): p. 1202-17.

2. P. C. Austin, *Balance diagnostics for comparing the distribution of baseline covariates between treatment groups in propensity-score matched samples.* Stat Med, 2009. **28**(25): p. 3083-107.
